# Servant Leadership Through a Gendered Lens: Role Congruity, Warmth–Competence Perceptions, and Leader Effectiveness

**DOI:** 10.3390/bs16071244

**Published:** 2026-07-21

**Authors:** Ge Cao, Guoyang Zheng

**Affiliations:** 1 Faculty of Engineering, The University of Sydney, Sydney, NSW 2006, Australia; caogesydney@163.com; 2 Faculty of Psychology, Shandong Normal University, Jinan 250014, China

**Keywords:** servant leadership, leader gender, role congruity theory, leader effectiveness

## Abstract

This study pivots from the universally beneficial view of servant leadership and examines how leader gender shapes its effectiveness evaluations through followers’ warmth and competence perceptions. Combining role congruity theory and the stereotype content model, we propose a congruity trap model and suggest that servant leadership produces different effectiveness evaluations depending on leader gender. Specifically, we hypothesize that servant leadership interacts with leader gender to produce divergent warmth and competence perceptions, and these perceptions in turn lead to perceived leadership effectiveness. In a 2 × 2 between-subjects online experiment (Study 1; *N* = 298), we found that servant leadership and leader gender interacted to predict both warmth and competence perceptions as hypothesized. In a three-wave time-lagged field study with full-time employees in China (Study 2; *N* = 194), we obtained partial support that the servant leadership and leader gender interaction was significant for competence perceptions but not for warmth perceptions. Gender-contingent differences in perceived effectiveness were more clearly transmitted through the indirect pathway involving competence. Taken together, these findings suggest that the competence perceptions constitute the more consistent mechanism through which servant leadership behaviors produce divergent evaluation outcomes for female and male leaders. Overall, our model and findings reveal that the benefits of servant leadership are not equally available to all leaders, illuminating how gender stereotypes create divergent evaluation outcomes from identical leadership behaviors.

## 1. Introduction

One of the most celebrated findings in contemporary leadership research is that servant leadership, which prioritizes followers’ needs, demonstrates empathy, and empowers others, consistently enhances leadership effectiveness across diverse organizational contexts (Eva et al., 2019; Liden et al., 2008). This consensus stems from an impressive array of empirical studies, reviews, and meta-analyses, each concluding that servant leadership predicts positive outcomes including follower performance, organizational citizenship behavior, and team effectiveness (Eva et al., 2019; Lee et al., 2020). As a result, the superiority of this approach has become accepted doctrine within academic scholarship (van Dierendonck, 2011), organizational practice (Sendjaya & Sarros, 2002), and leadership development programs (Greenleaf Center for Servant Leadership, 2016).

Critically, servant leadership’s effectiveness stems from its emphasis on communal, relational qualities—including care, empathy, interpersonal sensitivity, and concern for others’ well-being (Liden et al., 2008; van Dierendonck, 2011). These behavioral emphases distinguish servant leadership from other leadership approaches and align with evolving workplace values emphasizing collaboration and employee development (Parris & Peachey, 2013). Yet research on gender and leadership has established that communal characteristics are stereotypically associated with women and agentic characteristics with men (Eagly & Karau, 2002; Heilman, 2012), while leadership prototypes remain predominantly masculine in character (Koenig et al., 2011; Schein, 2001). Scholars have documented the backlash female leaders face for agentic behavior (Rudman et al., 2012), the competence skepticism they encounter when behaving communally (Heilman, 2012), and the double bind these dynamics jointly create (Eagly & Karau, 2002). What this literature leaves underspecified, however, is the evaluative process by which gender stereotypes shape the reception of servant leadership. Role congruity theory identifies that communal female leaders face competence skepticism, but it does not specify the perceptual pathway through which this skepticism is produced, nor does it generate predictions about what happens when male leaders adopt communal leadership styles. Hence, we know far less about how leader gender fundamentally alters the evaluative meaning of servant leadership behaviors for both men and women.

Servant leadership is distinguished from other relational leadership approaches by its primary orientation toward serving the interests of followers and broader stakeholders rather than achieving organizational performance goals (Greenleaf, 1977; Liden et al., 2008). By comparison, transformational leadership, while incorporating relational elements such as individualized consideration, foregrounds inspirational motivation and idealized influence, both of which center on mobilizing followers toward a leader-defined vision (Bass & Avolio, 1994). Ethical leadership and authentic leadership similarly retain an agentic core: the former through normative standard-setting and moral compliance modeling (Brown et al., 2005), the latter through emphasis on the leader’s own authenticity and self-concept (Walumbwa et al., 2008). Servant leadership is not exclusively communal; it maintains concern for organizational effectiveness and ethical stewardship (Liden et al., 2008; van Dierendonck, 2011). Yet, its defining emphasis on follower welfare and stakeholder service gives it a communal character that is both more fundamental and more consistent than in other leadership frameworks. Altogether, servant leadership’s predominantly communal focus suggests that leader gender, through its alignment with or violation of communal gender stereotypes, is likely to create meaningful differences in how servant leadership behaviors are evaluated.

To investigate this question, we integrate role congruity theory (Eagly & Karau, 2002) and the stereotype content model (Fiske et al., 2002; Cuddy et al., 2008). For female leaders, servant leadership’s communal behaviors align with gender-role expectations and are accordingly read as confirmation of expected traits rather than evidence of deliberate leadership choices, reducing their informational value for leadership evaluation (Biernat & Kobrynowicz, 1997). This congruity trap activates the stereotype content model’s warmth–competence tradeoff: servant leadership enhances warmth perceptions while simultaneously triggering compensatory reductions in competence perceptions (Cuddy et al., 2008). For female leaders, this pattern suggests a constrained evaluative trajectory: their communal behaviors may contribute little to competence perceptions, an important criterion against which leadership effectiveness is judged in organizational contexts.

**Figure 1:**
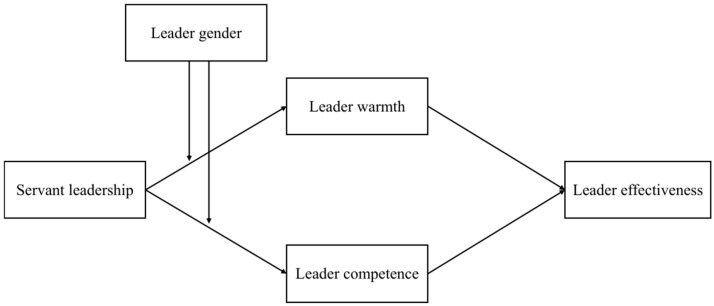
The theoretical model.

For male leaders, the same servant leadership behaviors produce an opposite pattern. Servant leadership’s communal emphasis directly contradicts the agentic norms associated with masculine leadership (Koenig et al., 2011), creating an expectancy violation that operates in male leaders’ favor. Research indicates that positive behaviors deviating from role-based expectations elicit stronger and more favorable evaluations than expected behaviors, as violations signal genuine personal qualities more convincingly than role-consistent behavior can (Bettencourt et al., 1997). This expectancy violation amplifies warmth perceptions for male servant leaders. Meanwhile, male leaders’ higher baseline status and competence presumptions in organizational settings (Foschi, 2000) buffer the warmth–competence tradeoff, allowing warmth gains without equivalent competence losses (Cuddy et al., 2008). Male servant leaders thus achieve more favorable warmth and competence profiles, which constitute the primary perceptual pathways through which servant leadership’s gendered evaluative consequences translate into differential effectiveness judgments. The theoretical model is shown in Figure 1.

This research makes several important theoretical contributions to the extant literatures on servant leadership, role congruity theory, and the stereotype content model. First, by examining leader gender as a boundary condition of servant leadership’s evaluative consequences, we challenge the prevailing consensus that servant leadership is a universally beneficial leadership style (Eva et al., 2019; Lee et al., 2020). We offer a framework for understanding when and for whom its benefits are more or less available, thus providing a more differentiated and contextually sensitive account of servant leadership’s effects than average-effect summaries have thus far captured (Hoch et al., 2018). Second, we extend role congruity theory by specifying a perceptual mechanism through which stereotype-congruent communal behavior may generate evaluative disadvantage for female leaders—not through backlash for norm violation, but through attribution discounting that reduces the diagnostic value of communal behaviors for leadership effectiveness. This extends the double bind beyond the incongruity case and identifies congruity itself as a potential source of evaluative constraint. Third, we contribute to the stereotype content model by examining how gendered expectancy congruity moderates the warmth and competence inferences that servant leadership generates. By integrating the model with role congruity logic in an organizational leadership context, we aim to specify the conditions under which servant leadership behaviors produce differential perceptions and effectiveness outcomes—depending on who enacts them.

## 2. Theoretical Background and Hypotheses

### 2.1. Servant Leadership

Servant leadership is defined by the priority the leader places on serving others. Greenleaf (1977), who coined the concept, described the servant leader as one for whom serving comes first and leading comes second, directing attention not toward personal advancement or organizational goals but toward the growth, well-being, and development of those served. Subsequent theoretical work operationalized this orientation into behaviorally distinct dimensions: putting followers’ interests first, helping others grow and succeed, emotional healing, creating value for the community, and empowering followers (Liden et al., 2008; van Dierendonck, 2011). Together, these dimensions reflect what Lemoine et al. (2019) identified as servant leadership’s distinctive moral foundation, a consequentialist commitment to benefiting multiple stakeholders, from followers to customers to broader communities.

What distinguishes servant leadership from other prominent leadership styles is not merely that it incorporates communal behaviors, but that communal orientation is constitutive of the construct rather than instrumental to it. Transformational leaders may exhibit warmth, support, and individualized consideration, but these behaviors serve as means to motivate followers toward collective goals and a shared vision (Bass, 1985). The theoretical core of transformational leadership remains agentic: it centers on the leader’s capacity to articulate direction, generate enthusiasm, and catalyze change. Servant leadership, by contrast, is organized around the subordination of the leader’s own agenda to the needs and development of others. Placing followers first is not a strategy servant leaders selectively deploy; it is what servant leadership is.

This theoretical distinction matters for the present study. Because the behaviors constitutive of servant leadership, including nurturing, supporting, empowering, and prioritizing others’ needs above one’s own, overlap substantially with the communal qualities that gender stereotypes prescriptively associate with women (Eagly & Karau, 2002), it becomes theoretically important to ask whether servant leadership’s communal foundation interacts with leader gender in shaping how effectiveness is perceived. Among leadership styles, servant leadership provides the clearest test of this question precisely because its communal character is not peripheral or stylistic, but built into its definition.

### 2.2. Theoretical Background

Role congruity theory (Eagly & Karau, 2002) proposes that gender bias in leadership evaluation arises from perceived incongruity between the communal qualities prescriptively associated with women and the agentic qualities prescriptively associated with leadership. This structural mismatch produces two well-documented forms of prejudice. Women are seen as less naturally suited for leadership because they are presumed to lack the assertiveness and dominance that leadership roles demand. When women do display agentic leadership behaviors, they face social penalties for violating communal gender prescriptions, a pattern extensively documented as backlash (Rudman et al., 2012). The theory thus accounts for a substantial share of the gender disparities observed in leadership evaluation and selection.

Yet role congruity theory’s focus on agentic behavior and stereotype violation leaves the perceptual mechanism underlying these evaluative outcomes underspecified. The theory identifies that gender shapes leadership evaluation and predicts that role incongruity produces negative outcomes, but it does not fully explain how evaluators’ multidimensional impressions of a leader are actually constructed. When someone evaluates a leader, they are not merely checking for role consistency; they are forming judgments along multiple dimensions simultaneously. Understanding this process requires a complementary framework.

The stereotype content model (Fiske et al., 2002; Cuddy et al., 2008) provides this framework. The model proposes that perceivers organize their impressions of individuals and groups along two fundamental dimensions: warmth, capturing perceived intentions, trustworthiness, and interpersonal orientation, and competence, capturing perceived ability, skill, and effectiveness. These dimensions are not independent: perceivers who form high warmth impressions of a target tend to form relatively lower competence impressions, and vice versa. This compensatory tradeoff reflects motivated social cognition rather than accurate assessment, and it operates consistently across group-level and individual-level judgments (Judd et al., 2005; Kervyn et al., 2009).

For understanding gender bias in leadership, the warmth–competence tradeoff is theoretically significant because it specifies how preexisting gender stereotypes shape the interpretation of behavioral evidence. Women as a social category are stereotypically positioned as high in warmth and lower in competence; men are positioned as high in competence and lower in warmth (Eagly & Mladinic, 1994; Fiske et al., 2002). When a leader engages in communal, warmth-generating behaviors, these behaviors are not processed in a stereotype-free environment. They interact with the gender-based warmth and competence expectations that perceivers already hold, and the resulting dimensional judgments may diverge substantially from what the behavior itself would suggest.

### 2.3. Hypotheses Development

#### 2.3.1. Servant Leadership, Leader Gender, and Perceived Warmth

Servant leadership behaviors, including attending to followers’ emotional needs, prioritizing their development over personal interests, and demonstrating sustained empathy, are among the most direct behavioral expressions of interpersonal warmth. The stereotype content model defines warmth as perceived benevolent intentions, trustworthiness, and interpersonal orientation (Fiske et al., 2002; Cuddy et al., 2008), a description that maps closely onto servant leadership’s defining dimensions such as emotional healing, putting followers first, and creating value for the community (Eva et al., 2019; Liden et al., 2008). Leaders who consistently subordinate their own interests to followers’ needs and who offer support through difficulty provide observers with sustained behavioral evidence of benevolent intent. We expect, therefore, that servant leadership is positively associated with perceived warmth regardless of the leader’s gender.

The question is whether this warmth enhancement is equally strong for female and male leaders. Meta-analytic evidence confirms that leader stereotypes remain predominantly masculine, meaning that communal qualities are associated with women but not with the leadership role itself (Koenig et al., 2011). Role congruity theory adds that women are prescriptively expected to be warm, nurturing, and interpersonally attentive (Eagly & Karau, 2002), so a woman who enacts servant leadership is doing something observers already anticipated. The informational value of confirmatory behavior is limited: it updates no prior expectation and provides no new evidence about this individual leader’s intentions beyond what gender already implied. These communal behaviors tend to be absorbed into the existing gender category, attributed to dispositional femininity rather than to deliberate leadership orientation (Heilman, 2012). The warmth signal is real but incrementally small.

When men enact the same behaviors, the perceptual dynamic changes. Prescriptive masculine stereotypes emphasize assertiveness, task-orientation, and agency (Eagly & Karau, 2002; Koenig et al., 2011), and communal servant leadership behaviors run against these expectations. Research consistently shows that men who violate masculine gender norms are evaluated differently from women performing identical behaviors, because the violation itself demands explanation (Moss-Racusin et al., 2010; Rudman et al., 2012). When behavior contradicts prior expectations, perceivers assign it greater diagnostic weight precisely because it cannot be attributed to gender-based dispositions (Bettencourt et al., 1997). A male leader who demonstrates empathy and prioritizes follower development is doing something the gender stereotype cannot account for; observers must therefore attribute it to something distinctive about this particular leader’s orientation. The result is a stronger and more personally grounded warmth attribution than women performing identical behaviors would typically receive.

**Hypothesis** **1a.** *The relationship between servant leadership and perceived leader warmth is moderated by leader gender, such that servant leadership is more positively associated with perceived warmth for male leaders than for female leaders*.

#### 2.3.2. Servant Leadership, Leader Gender, and Perceived Competence

The stereotype content model’s compensatory logic suggests that the warmth enhancement associated with servant leadership does not come without cost. When a target’s warmth perceptions increase, competence perceptions tend to decrease correspondingly, as perceivers draw on implicit assumptions about resource constraints to calibrate their judgments across the two dimensions (Cuddy et al., 2008; Judd et al., 2005). Servant leadership, by consistently signaling interpersonal warmth and other-orientation, may therefore activate this tradeoff, placing downward pressure on competence perceptions even as it elevates warmth. Whether the resulting competence penalty is meaningful, however, depends on where in the warmth–competence perceptual space the leader is positioned to begin with.

The stereotype content model documents that social groups cluster in distinct regions of this space: women are stereotypically associated with high warmth and lower competence, while men are associated with higher competence and lower warmth (Fiske et al., 2002; Eagly & Mladinic, 1994). This structural asymmetry shapes how the warmth–competence tradeoff plays out for female versus male servant leaders. For women, servant leadership behaviors amplify a warmth profile that is already high relative to competence. The compensatory mechanism pushes their judgment further toward the low-competence end of the space, reinforcing the gendered stereotype already in place (Heilman, 2012; Koenig et al., 2011). Each additional warmth cue does not simply trigger a generic tradeoff; it also activates the group-level stereotype, lending it additional force. Female servant leaders therefore face a convergence of the individual-level compensation mechanism and the group-level stereotype, both operating in the same direction.

For male leaders, the same compensatory logic applies in principle, but its effects are substantially attenuated by the starting position. Masculine stereotypes associate male leaders with competence, decisiveness, and strategic capability (Eagly & Karau, 2002; Heilman, 2012; Koenig et al., 2011), creating a high competence baseline from which the warmth–competence tradeoff must displace them. This displacement is unlikely to produce a meaningful penalty. More importantly, however, the expectancy-violating nature of men’s communal behavior introduces a further dynamic. Because a male leader’s warmth is unexpected, it cannot be dismissed as dispositional; perceivers instead attribute it to deliberate, intentional choice (Bettencourt et al., 1997). A leader who, from a position of presumed competence, actively chooses to prioritize follower development, extend empathy, and subordinate personal recognition to collective growth signals something about leadership sophistication that stereotype-congruent behavior cannot. This deliberate investment in others, precisely because it violates masculine norms, reads as evidence of secure, confident leadership rather than as a competence deficit. The result is that servant leadership’s communal behaviors, rather than triggering a warmth–competence tradeoff for men, may actually enhance competence perceptions by signaling intentional, capable leadership.

**Hypothesis** **1b.** *The relationship between servant leadership and perceived leader competence is moderated by leader gender, such that servant leadership is more positively associated with perceived competence for male leaders than for female leaders*.

#### 2.3.3. Warmth, Competence, and Perceived Leader Effectiveness

Leadership effectiveness, as examined here, refers to followers’ perceptual judgments about how well leaders perform their role. This focus on perceived rather than actual effectiveness is theoretically grounded: the stereotype content model is a theory of social perception, and its predictions concern how observers evaluate targets based on the impressions they form through interaction rather than through objective performance records (Fiske et al., 2002). In organizational settings, these evaluative judgments shape leaders’ authority, their followers’ willingness to be led, and their broader reputational standing, and they do so through processes of social perception that operate independently of objective outcomes. Perceived effectiveness is therefore not merely a proxy for actual effectiveness; it is the construct most directly connected to the social-evaluative dynamics the stereotype content model describes.

Warmth perceptions should contribute to perceived effectiveness because they shape followers’ fundamental orientation toward the leader. When followers perceive a leader as warm, they infer that the leader’s motivations are oriented toward their interests rather than toward self-serving ends, and this inference extends the trust that makes leadership influence possible (Cuddy et al., 2008). A leader judged as genuinely caring is one whose direction followers are willing to accept, whose decisions they are inclined to interpret charitably, and whose role they are motivated to support. These relational dynamics are not peripheral to leadership; they constitute the social foundation through which leaders exercise influence. Warmth perceptions thus contribute to effectiveness evaluations not through a direct inference of capability, but by establishing the interpersonal conditions under which leadership is judged as working.

Competence perceptions contribute through a different but equally consequential pathway. Leadership roles are defined in part by task accomplishment, goal attainment, and the capacity to guide followers and organizations toward desired outcomes. Because leadership prototypes in organizational contexts are organized predominantly around agentic, capability-related qualities (Eagly & Karau, 2002; Koenig et al., 2011), followers who attribute high competence to their leaders are more likely to view them as fulfilling what effective leadership requires.

**Hypothesis** **2a.** *Perceived leader warmth positively relates to perceived leader effectiveness*.

**Hypothesis** **2b.** *Perceived leader competence positively relates to perceived leader effectiveness*.

The hypotheses developed above specify two sets of relationships: leader gender moderates the effects of servant leadership on warmth and competence perceptions (H1a, H1b), and warmth and competence perceptions in turn predict perceived effectiveness (H2a, H2b). Together, these relationships constitute a moderated mediation model in which warmth and competence serve as the perceptual pathways through which servant leadership’s gendered evaluative consequences translate into overall judgments of leader effectiveness.

**Hypothesis** **3a.** *Perceived leader warmth mediates the interaction effect of servant leadership and leader gender on perceived leader effectiveness, such that the indirect effect through warmth is more positive for male leaders than for female leaders*.

**Hypothesis** **3b.** *Perceived leader competence mediates the interaction effect of servant leadership and leader gender on perceived leader effectiveness, such that the indirect effect through competence is more positive for male leaders than for female leaders*.

## 3. Materials and Methods

We tested the proposed model using a multimethod approach that integrates a preregistered online experiment (Study 1) with a three-wave field study (Study 2). This strategy was designed to maximize both internal and external validity. We conducted both studies in mainland China. We treat this context as theoretically pertinent rather than merely sampling-convenient, for five reasons. First, leadership prototypes in China remain predominantly masculine, making gender a salient lens through which communal leadership behaviors are evaluated. Meta-analytic evidence documents a robust “think manager–think male” association across cultures (Koenig et al., 2011; Schein, 2001), and cross-national research confirms that leader stereotypes associating leadership with agentic qualities are not confined to Western settings (Koenig et al., 2011). The GLOBE program further documents that Chinese societal leadership ideals combine assertiveness and performance orientation with collectivist relational values (House et al., 2004). This configuration is directly consequential for our model: examining how followers evaluate servant leadership’s communal behaviors when enacted by female versus male leaders is central to testing whether such behaviors are interpreted differently depending on whether they confirm or violate gender-based expectations. Second, the warmth–competence framework underlying our hypotheses has been validated cross-culturally, including in East Asian contexts. Cross-cultural research demonstrates that the warmth–competence structure generalizes beyond Western samples (Cuddy et al., 2008; Durante et al., 2017), and research in Chinese samples specifically documents that gender and social role expectations shape warmth and competence attributions in ways consistent with our theorizing (Hu et al., 2022). China is therefore not a context in which our theoretical constructs lack meaning; it is one in which gendered warmth–competence dynamics have been empirically observed. Third, China combines high female labor force participation with persistent underrepresentation of women in senior leadership roles (International Labour Organization, 2019; World Economic Forum, 2023). This pattern is precisely what role congruity theory predicts would sustain gender-contingent interpretation of leadership behavior: women’s communal conduct may be read as role-consistent rather than as evidence of leadership capability, whereas men’s communal conduct carries greater diagnostic weight (Eagly & Karau, 2002; Heilman, 2012). The organizational stakes of gendered leadership evaluation thus remain high in this context. Fourth, relational forms of leadership carry deep conceptual roots in Chinese organizational contexts, emphasizing benevolence, relational sensitivity, and moral example (Cheng et al., 2004). Servant leadership has been linked to positive follower outcomes in Asian organizational settings (Chen et al., 2015). What this literature has not addressed is whether the evaluative benefits of communal, service-oriented leadership accrue equally to female and male leaders when gender stereotypes shape how such behaviors are interpreted. Our study speaks directly to that gap. Fifth, and perhaps most importantly, China constitutes a theoretically stringent rather than permissive test of our hypotheses. As Hofstede’s (2001) cultural dimensions research and the GLOBE study (House et al., 2004) document, Chinese cultural values combine strong collectivist relational orientations with persistent gender-differentiated role expectations, meaning that communal prescriptions for women and agentic expectations for men remain highly salient. We do not claim that China is the only appropriate setting for this research, nor do we treat our findings as automatically generalizable; we address cross-cultural replication explicitly in limitations.

Study 1 employed a controlled experimental design to establish causal evidence that leader gender moderates the effects of servant leadership on perceived warmth and competence. By randomly assigning participants to manipulated conditions, Study 1 allows us to rule out third-variable confounds and draw directional inferences about how servant leadership behaviors, in combination with leader gender cues, shape dimensional social perceptions. Study 2 then extended these findings to naturalistic organizational settings, using a time-lagged survey design to replicate the interaction effects of servant leadership and leader gender, while additionally testing the full moderated mediation model—specifically whether warmth and competence serve as mechanisms linking the interaction term of servant leadership and leader gender to leader effectiveness evaluations. Together, the two studies provide complementary evidence. Study 1 demonstrated that servant leadership behaviors generate gender-contingent perceptual responses under controlled conditions, while Study 2 tested whether these perceptions extend to real organizational contexts and whether warmth and competence perceptions mediate the relationship between the servant leadership–gender interaction and downstream effectiveness evaluations. Both studies were conducted in accordance with standard research ethics principles. Participation was voluntary, all participants provided informed consent prior to participation, no personally identifying information was collected, participants were informed of their right to withdraw at any time without consequence, and all data were stored securely and anonymously.

### 3.1. Study 1

#### 3.1.1. Participants

We conducted a preregistered study (https://aspredicted.org/tu7r3u.pdf, accessed on 25 June 2026) with full-time employees from Credamo (https://www.credamo.com/home.html#/, accessed on 1 March 2023), a widely used online survey platform in China equivalent to MTurk and Prolific, to participate in exchange for ¥3. Participants were presented with an invitation letter that included information about the study (e.g., duration, procedure, and compensation). Participation was voluntary, and respondents were assured that their personal information would be kept confidential. The sample size for this study was determined through statistical power analysis using G*Power software 3.1 (Faul et al., 2007, 2009). Assuming a medium between-conditions effect size of d = 0.25, 269 participants were required to achieve an α level of 0.05 and statistical power of 1 − β = 0.80. To account for potential missing data, we predetermined a recruitment target of 75 participants per condition.

Therefore, we invited 300 full-time employees to participate in a between-subjects experiment (servant leadership: high vs. low; leader gender: female vs. male), and participants were randomly assigned to one of the four experimental conditions. We retained only those participants who passed the attention check, resulting in a final sample of 298 participants (75 in the high female servant leadership condition, 74 in the low female servant leadership condition, 74 in the high male servant leadership condition, and 75 in the low male servant leadership condition; 63.0% male; Mage = 36.45, SDage = 8.42).

#### 3.1.2. Procedure and Experimental Design

We employed a 2 (servant leadership: high vs. low) × 2 (leader gender: female vs. male) between-subjects experimental design. Participants were randomly assigned to one of four conditions. After providing informed consent, participants read a scenario describing a supervisor at a consulting company’s project team. The scenario presented background information about a small project team consisting of the supervisor and three members, and described a workplace context that participants had purportedly been part of for six months. Participants were instructed to carefully read the scenario and imagine themselves as a direct witness to the described events, forming impressions as realistically as possible. Following the scenario, participants completed manipulation checks, rated their perceptions of the leader’s warmth and competence. The survey concluded with demographic questions and an attention check. Finally, participants were debriefed about the study’s purpose and thanked for their participation.

**Manipulation.** Participants read a scenario of servant leadership manipulation (see Appendix A), used in Wu et al. (2021). The manipulation of servant leadership was designed by following the methods from previous experimental research on servant leadership (e.g., Heine et al., 2023; Song et al., 2024; van Dierendonck et al., 2014).

Leader gender was manipulated through the manager’s name and corresponding pronouns in the scenario. In the female leader conditions, the manager was named “Li Ting” with female pronouns (she/her). In the male leader conditions, the manager was named “Li Gang” with male pronouns (he/him). All other aspects of the scenarios remained identical across gender conditions. We selected ethnically ambiguous names to minimize potential confounds related to racial stereotypes.

#### 3.1.3. Measures

**Leader warmth and competence.** We measured perceptions of leader warmth using the five-item warmth subscale from Fiske et al.’s (2007) Stereotype Content Model. Participants rated the extent to which they perceived the manager as warm, good-natured, tolerant, sincere, and friendly (1 = strongly disagree, 7 = strongly agree). In this study, Cronbach’s α = 0.93.

Leader competence was measured using the five-item competence subscale from Fiske et al.’s (2007) Stereotype Content Model. Participants rated the extent to which they perceived the manager as competent, confident, capable, efficient, and intelligent (1 = strongly disagree, 7 = strongly agree). In this study, Cronbach’s α = 0.91.

**Manipulation check.** To verify the effectiveness of our servant leadership manipulation, we measured participants’ perceptions of the leader’s servant leadership using the seven-item scale (Liden et al., 2008). Participants rated their agreement with statements such as “This manager makes subordinates’ career development a priority” (1 = strongly disagree, 7 = strongly agree). This shortened scale has demonstrated strong psychometric properties and high convergent validity with the full 28-item measure (α > 0.80). In this study, Cronbach’s α = 0.96.

#### 3.1.4. Results

The manipulation check confirmed that participants in the high servant leadership condition perceived significantly higher servant leadership behaviors (M = 5.46, SD = 0.78) than those in the low servant leadership condition (M = 2.81, SD = 0.81), F(1, 296) = 832.71, *p* < 0.001.

**Figure 2:**
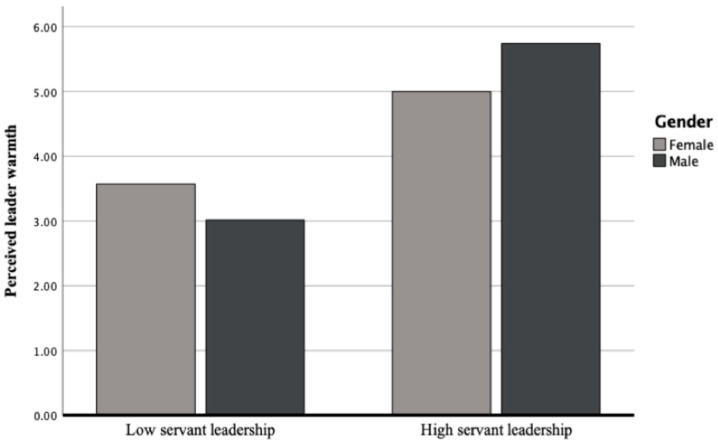
The interaction effect of servant leadership and leader gender on perceived leader warmth in Study 1.

A 2 × 2 ANOVA on leader warmth revealed a significant main effect of servant leadership, F(1, 294) = 461.56, *p* < 0.001, with high servant leadership leaders (M = 5.36, SD = 0.88) perceived as warmer than low servant leadership leaders (M = 3.30, SD = 0.90). The main effect of leader gender was not significant, F(1, 294) = 0.67, *p* = 0.42. The interaction was significant, F(1, 294) = 45.68, *p* < 0.001 (see Figure 2). For female leaders, high servant leadership (M = 5.00, SD = 0.81) was perceived as warmer than low servant leadership (M = 3.59, SD = 0.85), F(1, 147) = 107.15, *p* < 0.001. Similarly, for male leaders, high servant leadership (M = 5.73, SD = 0.78) was perceived as warmer than low servant leadership (M = 3.02, SD = 0.86), F(1, 147) = 403.62, *p* < 0.001. Critically, the beneficial effect of servant leadership on perceived warmth was significantly stronger for male leaders than for female leaders, supporting Hypothesis 1a.

**Figure 3:**
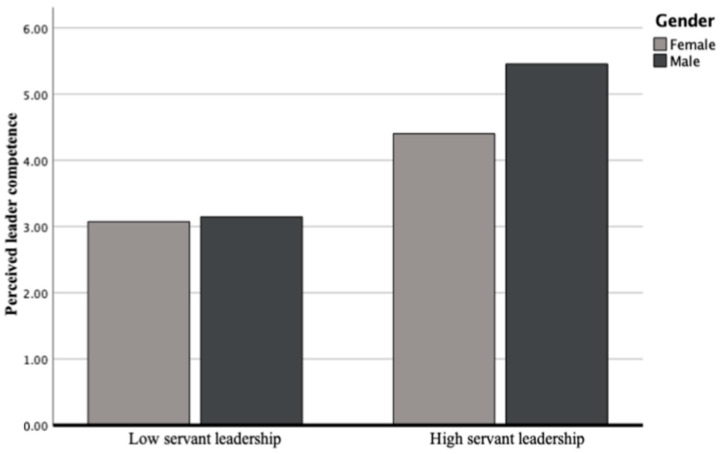
The interaction effect of servant leadership and leader gender on perceived leader competence in Study 1.

A 2 × 2 ANOVA on leader competence revealed a significant main effect of servant leadership, F(1, 294) = 487.29, *p* < 0.001, suggesting that servant leadership behaviors significantly influence competence perceptions. The main effect of leader gender was significant, F(1, 294) = 46.63, *p* < 0.001. The interaction effect was significant, F(1, 294) = 35.60, *p* < 0.001 (see Figure 3). For female leaders, high servant leadership (M = 4.40, SD = 0.70) was perceived as higher competence than low servant leadership (M = 3.08, SD = 0.80), F(1, 147) = 115.20, *p* < 0.001. Similarly, for male leaders, high servant leadership (M = 5.46, SD = 0.72) was perceived as higher competence than low servant leadership (M = 3.15, SD = 0.61), F(1, 147) = 449.90, *p* < 0.001. Thus, the beneficial effect of servant leadership on perceived competence was significantly stronger for male leaders than for female leaders, supporting Hypothesis 1b.

#### 3.1.5. Discussion

Study 1 offered initial support for our hypotheses that leader gender moderates the perceptions of warmth and competence regarding servant leadership. These results provide initial support for the causal role of the interaction effects of servant leadership and leader gender. However, we did not test whether warmth and competence perceptions affect leader effectiveness. Building on this initial examination, we aimed to provide stronger evidence in a field study that allows comprehensive tests of our theoretical model.

### 3.2. Study 2

#### 3.2.1. Sample and Procedure

Study 2 employed a three-wave time-lagged design to reduce common method bias and establish temporal separation between the predictor and outcome variables. Participants were full-time employees recruited from the authors’ research participant pool, comprising individuals from a range of industries and organizational types who had previously expressed willingness to take part in organizational research studies. All participants were employed full-time at the time of data collection and were asked to evaluate their current direct supervisor. The first-round survey was conducted in March 2023, the second-round survey was conducted in April 2023, and the third-round survey was conducted in May 2023. Before we distributed the first-round questionnaire, we assured participants of confidentiality and voluntary participation and stated that the surveys would be used solely for academic research. Participants received ¥7, ¥10, and ¥15 for the first-, the second-, and the third-round survey, respectively.

Our initial sample was 300 employees. At Time 1, participants completed questionnaires regarding demographics, servant leadership, and leader gender. At Time 2, participants completed questionnaires regarding perceived leader warmth and competence. At Time 3, participants completed questionnaires regarding their perceived leader effectiveness. A total of 300 full-time employees completed Wave 1 of the survey. Of these, 238 also completed Wave 2, and 194 completed all three waves, yielding an overall attrition rate of 35.3% from Wave 1 to Wave 3. Because participant loss across waves raises the possibility of non-random attrition, we conducted attrition analyses to examine whether employees who completed all three waves differed systematically from those who dropped out. We compared completers (*n* = 194) with non-completers (*n* = 106) on all Wave 1 variables, including follower gender, follower age, education, organizational tenure, tenure with the current supervisor, leader gender, and Wave 1 servant leadership ratings. For continuous variables, we used independent-samples *t*-tests; for categorical variables, we used chi-square tests. None of the Wave 1 comparisons reached conventional levels of statistical significance (all *p*s > 0.05). Completers and non-completers did not differ significantly in Wave 1 servant leadership perceptions (M = 4.53 vs. 4.58, respectively; *p* = 0.76), follower age (M = 31.90 vs. 33.19; *p* = 0.22), and leader gender, χ^2^(1) = 0.75, *p* = 0.39. We also examined whether participants who dropped out after Wave 2 (n = 44) differed from completers on Wave 2 warmth and competence ratings. These comparisons were likewise nonsignificant (warmth: M = 3.84 vs. 3.77, *p* = 0.56; competence: M = 3.33 vs. 3.28, *p* = 0.54). Taken together, these results suggest that attrition across waves was unlikely to be systematically related to the demographic characteristics or substantive study variables measured at earlier waves.

Formal analyses were restricted to the 194 participants who completed all three waves. Each participant evaluated their own direct supervisor independently, with no two participants sharing the same leader, yielding 194 independent leader-follower dyads. The data therefore contain no hierarchical nesting structure, and multilevel modeling was not required. A post hoc sensitivity analysis indicates that with *N* = 194, the study achieved adequate statistical power (1 − β > 0.80, α = 0.05) to detect small-to-medium interaction effects (f^2^ ≥ 0.06) in the moderated mediation analyses.

#### 3.2.2. Measures

All measures were translated into Chinese by authors using the back–translation procedures recommended by Brislin (1986).

**Servant leadership.** As in Study 1, servant leadership was measured using the 7-item scale developed by Liden et al. (2008). In this study, Cronbach’s α = 0.95.

**Leader warmth and competence.** As in Study 1, perceived leader warmth and competence were measured using the five-item warmth subscale and the five-item competence subscale from Fiske et al.’s (2007) Stereotype Content Model. In this study, Cronbach’s α = 0.90 for perceived leader warmth and Cronbach’s α = 0.93 for perceived leader competence.

**Leader effectiveness.** Leadership effectiveness was measured using van Knippenberg and van Knippenberg (2005)’s four-item scale. A sample item is “This manager is an effective leader” (1 = strongly disagree, 7 = strongly agree). This measure has been validated in multiple organizational studies and demonstrates strong predictive validity for objective leadership outcomes. In this study, Cronbach’s α = 0.91.

**Control variables.** We collected demographic information including participant age, gender and work experience (in years) as covariates in analyses.

#### 3.2.3. Results

Before testing the hypotheses, we used Mplus 8.6 to conduct confirmatory factor analyses (CFAs) to examine the distinctiveness of our primary study variables (i.e., servant leadership, leader warmth, leader competence, and leader effectiveness). As shown in Table 1, the four-factor model fit the data adequately (χ^2^ = 161.45, *df* = 183, CFI = 0.99, TLI = 0.98, RMSEA = 0.08, SRMR = 0.03) and better than the three-factor model (Δχ^2^ = 679.75, Δ*df* = 3, *p* < 0.001), the two-factor model (Δχ^2^ = 1154.90, Δ*df* = 5, *p* < 0.001) and the single-factor model (Δχ^2^ = 1686.40, Δ*df* = 6, *p* < 0.001). To test the proposed model, we used Process 4.1 to conduct a path analysis and construct bias-corrected bootstrap confidence intervals (CIs) around the estimated indirect effect.

Given that servant leadership, warmth, competence, and perceived effectiveness were all rated by the same source, we conducted an Unmeasured Latent Method Construct (ULMC) analysis in which all items were allowed to load on a latent method factor, in addition to their respective substantive factors. The method-factor model (χ^2^ = 162.06, df = 182, CFI = 0.98, TLI = 0.98, RMSEA = 0.09, SRMR = 0.03) did not fit the data substantially better than the baseline four-factor model (Δχ^2^(1) = 0.61, *p* > 0.05). Substantive path coefficients changed by less than 0.05 in magnitude after including the method factor, suggesting that common method bias did not materially distort the observed relationships.

**Figure 4:**
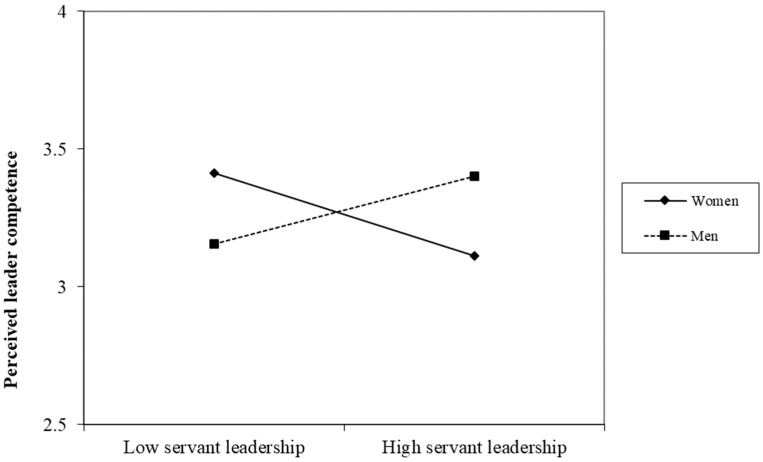
The interaction effect of servant leadership and leader gender on perceived leader competence in Study 2.

Descriptive statistics and correlations are presented in Table 2. Table 3 summarizes the results of path analyses. As shown in Table 3, the interaction term of servant leadership and leader gender in predicting perceived leader warmth was not significant, while its effect on perceived leader competence was significant (for perceived leader warmth: b = 0.08, *p* = 0.29; for perceived leader competence: b = 0.20, *p* < 0.001). As presented in Figure 4, simple slope tests confirmed that the relationship between servant leadership and perceived leader competence was significant and negative for female leaders (b = −0.11, *p* < 0.01), whereas it was significant and positive for male leaders (b = 0.09, *p* < 0.01). These findings support hypothesis 1b.

The relationship between perceived leader warmth and leader effectiveness was significant (b = 0.16, *p* < 0.01), and the relationship between perceived leader competence and leader effectiveness was significant (b = 0.15, *p* < 0.05), supporting hypotheses 2a and 2b. Monte Carlo simulations revealed that the conditional indirect effect of servant leadership on leader effectiveness via perceived leader competence was negative and significant for female leaders (effect size of indirect effect = −0.02, the 95% CI excluded zero [−0.04, −0.00]), and was positive and significant for male leaders (effect size of indirect effect = 0.01, the 95% CI excluded zero [0.00, 0.03]). These findings supported Hypothesis 3b.

#### 3.2.4. Discussion

The results of Study 2 provide partial support for the proposed model: the interaction effect of servant leadership and leader gender on leader effectiveness mediated by perceived leader competence. These findings suggest that there is no gender effect of perceived leader warmth. Study 2 replicated and extended the finding of Study 1 in the field context.

## 4. General Discussion

The divergence between Study 1 and Study 2 on the warmth pathway is worth examining directly. In Study 1, participants evaluated hypothetical leaders based on brief behavioral descriptions, with no prior relationship to draw on. In this context, gender stereotypes function as the primary lens through which servant leadership behaviors are interpreted, and the gender-contingent warmth effect emerged accordingly. Study 2 presents a different evaluative situation: followers assessed leaders they had worked with over time, bringing accumulated firsthand knowledge to their judgments. This distinction matters theoretically. Research on impression formation suggests that as individuating relationship knowledge accumulates, perceivers rely less on categorical group expectations and more on direct experience when forming trait judgments (Fiske & Neuberg, 1990). Warmth impressions in ongoing relationships are shaped substantially by interaction history, leaving less room for stereotype-based processing to differentiate male from female servant leaders. Competence perceptions behave differently: even in established relationships, followers continue to update their assessments of a leader’s judgment and capability based on observed behavior, keeping the gender-stereotypic interpretation of those behaviors consequential. That the competence moderation held in both studies while the warmth moderation did not suggests that the two pathways carry different contextual sensitivities, with the warmth pathway more dependent on the kind of categorical processing that novel evaluation settings naturally elicit.

### 4.1. Theoretical Contributions

First, this research contributes to the servant leadership literature by challenging the prevailing assumption that the style’s benefits are universal. Prior systematic reviews and meta-analyses have consistently documented positive average effects of servant leadership on follower and organizational outcomes, leading to an implicit consensus that servant leadership works across contexts (Eva et al., 2019; Lee et al., 2020). We challenge this conclusion by demonstrating that leader gender constitutes a meaningful boundary condition: across both studies, servant leadership behaviors generated more favorable competence perceptions for male leaders than for female leaders, and these competence differences translated into differential effectiveness evaluations through an indirect pathway. Rather than questioning whether servant leadership is beneficial on average, our findings reveal that its evaluative consequences are distributed asymmetrically depending on who enacts it—a qualification that average-effect summaries systematically obscure and that responds to calls for more systematic investigation of the conditions under which the style’s effects vary (Eva et al., 2019; Hoch et al., 2018).

Second, this research contributes to role congruity theory by revealing an evaluative mechanism through which stereotype conformity, not only stereotype violation, generates disadvantage for female leaders. Prior research has established that women face backlash when they display agentic behaviors that violate communal gender prescriptions (Eagly & Karau, 2002; Rudman et al., 2012; Williams & Tiedens, 2016), grounding the theory’s predictive account in a norm-violation logic. We extend this account by showing that communal behaviors congruent with gender expectations are discounted as evidence of leadership quality: because such behaviors are fully explained by gender-category membership, perceivers have no informational reason to attribute them to deliberate leadership orientation or individual capability (Biernat & Kobrynowicz, 1997; Heilman, 2012). By contrast, the same communal behaviors from male leaders contradict agentic expectations, demand an individual-level attribution, and thereby produce stronger competence inferences (Bettencourt et al., 1997)—a pattern directly reflected in the competence asymmetry we observed across both studies. These findings extend role congruity theory’s predictive scope by showing that evaluative disadvantage arises not only from incongruity through backlash, but also from congruity through discounting, identifying a second structural constraint on female leaders that the theory’s original framework did not fully anticipate.

Third, this research contributes to the stereotype content model by demonstrating that the warmth-competence tradeoff is not a fixed property of communal behavior but a process conditioned by expectancy congruity and evaluative context. Prior work has documented the tradeoff primarily at the group level and treated it as a relatively stable feature of social perception (Fiske et al., 2002; Cuddy et al., 2008; Fiske, 2018). We show that the tradeoff’s expression varies systematically: in Study 1, servant leadership elevated both warmth and competence for leaders of both genders, but the gains were significantly larger for male leaders whose communal behaviors violated agentic expectations—suggesting that expectancy violation amplifies the perceptual value of communal conduct rather than triggering a symmetric tradeoff. In Study 2, the competence asymmetry replicated while the warmth moderation did not, a divergence that points to a further contextual boundary: warmth judgments, readily formed from categorical cues, may yield to individuating relationship knowledge in ongoing organizational contexts, whereas competence judgments linked to agentic leadership criteria remain susceptible to gender-stereotypic processing throughout a working relationship (Fiske & Neuberg, 1990). Taken together, these findings suggest that the competence dimension is the more contextually robust channel through which gendered expectations shape leadership evaluation, and that both expectancy congruity and relational context determine which dimension carries greater perceptual primacy—an extension that complements prior theoretical work on the model’s organizational applications (Cuddy et al., 2011).

### 4.2. Practical Implications

The most direct practical implication concerns how organizations design leadership evaluation systems. If servant leadership behaviors generate systematically different effectiveness impressions depending on leader gender, then evaluation processes relying on global impressionistic judgments will tend to reproduce the biases we document. This is particularly consequential for promotion and advancement decisions, where cumulative evaluation disadvantages compound over time. Organizations would benefit from evaluation approaches that assess specific behavioral contributions rather than holistic effectiveness ratings, making visible what leaders actually did—whom they developed, which decisions they made, what outcomes followed—rather than relying on overall impressions susceptible to warmth-competence substitution. Evaluator training is equally important, but needs to go beyond general bias awareness. The mechanism at work here is not deliberate discrimination; it is the automatic application of warmth–competence tradeoff thinking to communal leadership behaviors. Training that makes this specific dynamic concrete, and that shows evaluators how identical behaviors can read differently depending on who enacts them, is more likely to produce evaluative change than broader diversity education alone.

The findings also speak to how organizations communicate about servant leadership, and to the difficult position female leaders occupy within current organizational realities. When servant leadership is framed primarily through warmth-oriented language—caring for employees, putting others first—it can inadvertently reinforce the association between communal leadership and diminished competence that female servant leaders already face. Presenting the style’s strategic and developmental dimensions with equal weight, emphasizing that servant leadership reflects deliberate investments in organizational capability rather than simply interpersonal warmth, may reduce this risk. At the individual level, female leaders operating in organizations that have not yet addressed these structural dynamics face a genuine dilemma: the behaviors that define effective servant leadership are the same behaviors that trigger competence skepticism in their case. We are deliberately cautious, however, about responding to this dilemma by advising women to compensate individually—for instance, by more visibly asserting competence while enacting communal behaviors. Such advice, however pragmatically motivated, shifts the burden of correcting a structural bias onto those who are already disadvantaged by it and does not address the evaluative conditions that produce the problem in the first place. The inequality our findings document originates in how leadership gets evaluated and communicated within organizations, and lasting change depends on addressing those conditions rather than on the additional compensatory work of individual female leaders.

### 4.3. Limits and Future Directions

Our empirical studies draw samples from Chinese organizational contexts, which may limit generalizability across cultural settings. China constitutes a theoretically pertinent setting for this research, given the salience of masculine leadership prototypes, the documented presence of gendered warmth–competence stereotypes in Chinese samples, and the high organizational stakes of gendered leadership evaluation in this context. At the same time, these features of the Chinese context may also shape the specific form and magnitude of the effects we observe. Confucian-influenced gender role prescriptions are historically strong in China, which we argued makes it a theoretically stringent test; it is possible that the competence discounting faced by female servant leaders is comparatively attenuated in cultural contexts where gender role boundaries are less sharply drawn, or alternatively amplified in contexts with stronger agentic leadership prototypes. Future research should replicate our model across cultural settings that vary systematically in gender egalitarianism, individualism–collectivism, and the strength of masculine leadership norms—such as individualistic Western European or North American contexts—to establish the cultural boundary conditions of our theory and to assess whether the competence pathway’s robustness holds across the range of contexts in which servant leadership is now practiced.

Second, a further limitation concerns the potential for common method bias in Study 2. Although we employed a three-wave time-lagged design to introduce temporal separation between the predictor, mediator, and outcome variables—and although our ULMC analysis indicated that a latent method factor accounted for negligible additional variance—Study 2 nonetheless relied on a single source, with followers rating all study variables including servant leadership, warmth, competence, and leader effectiveness. No statistical remedy, including the ULMC approach, can fully eliminate the possibility that shared method variance contributes to observed associations when all measures come from the same respondent. Future research would benefit from multi-source designs—for example, having followers rate servant leadership and warmth and competence perceptions while collecting effectiveness evaluations from supervisors, peers, or objective organizational records—to provide a more stringent and source-independent test of the proposed relationships.

A related limitation concerns temporal dynamics. Study 2’s three-wave design captures perceptions across approximately two months of measurement, but does not track how evaluative patterns evolve over the full arc of a leader–follower relationship. The divergence we observed between Study 1 and Study 2 on the warmth pathway—with gender-contingent warmth effects emerging under the novel evaluative conditions of the experiment but not in established field relationships—suggests that the relational duration and depth of prior interaction may themselves be important moderators of stereotype-based processing. Whether the competence discounting that female servant leaders experience attenuates as followers accumulate individuating information over longer relationships, or whether it persists and compounds, remains an open empirical question. Future research should employ longitudinal designs tracking warmth, competence, and effectiveness evaluations at multiple time points across the full course of supervisor–subordinate relationships (e.g., 1, 6, and 12 months of leader tenure), which would allow examination of whether the congruity trap is a durable feature of ongoing relationships or is most acute at earlier stages when categorical, stereotype-based processing predominates.

Third, our theoretical model does not consider how follower characteristics may moderate the gender-biased servant leadership effects. Followers’ own gender, gender role beliefs, implicit leadership theories, and previous experiences with female leaders may substantially influence how they evaluate servant leadership enacted by women versus men. For example, followers high in gender egalitarianism may be less likely to apply warmth–competence tradeoffs, whereas those holding traditional beliefs may exhibit stronger discounting. Future research should examine these follower-level moderators, testing whether individual differences in followers’ gender attitudes, leadership prototypes, or demographics buffer or amplify the biased evaluation patterns we document.

Fourth, a broader limitation concerns the partial support obtained for our theoretical model. Our hypothesized model proposed two parallel mediating pathways—warmth and competence—through which servant leadership’s gender-contingent evaluative consequences would translate into differential effectiveness judgments. The evidence supports the competence pathway consistently across both studies, but the warmth moderation effect emerged only in the experimental context of Study 1 and did not replicate in the field study of Study 2. The full dual-pathway model therefore remains without complete empirical support, and we caution against treating the warmth pathway as an established mechanism on the basis of the current evidence alone. Several boundary conditions deserve investigation in future research. The warmth pathway may be more operative in contexts that activate categorical, stereotype-based processing and may attenuate as perceivers accumulate individuating knowledge through ongoing interaction. Follower characteristics such as gender role attitudes, implicit leadership theories, and prior experience with female leaders may further moderate whether warmth perceptions are processed through a gender-stereotypic lens. Future research should directly test these boundary conditions to clarify when and for whom the warmth pathway constitutes an active mechanism in the gendered evaluation of servant leadership, and to establish whether the asymmetry between the two pathways reflects a stable feature of organizational evaluation or a context-sensitive one.

## 5. Conclusions

Drawing on role congruity theory and the stereotype content model, we found that servant leadership’s evaluative consequences are not gender-neutral. The evidence provides only partial support for our full model. In both studies, servant leadership interacted with leader gender to shape competence perceptions. These differences were linked to perceived effectiveness primarily through an indirect pathway involving competence, not through a robust direct effect on effectiveness. The warmth pathway was supported in the experiment but not in the field study. These findings suggest that competence perceptions are the more consistent mechanism behind gendered evaluations of servant leadership. We hope this work encourages future research on leader characteristics as boundary conditions and on how gender stereotypes shape the recognition of leadership quality.

## Figures and Tables

**Table 1:** Comparison of measurement in Study 2.

Model	χ^2^ (*df*)	CFI	TLI	RMSEA	Δχ^2^ (*df*)	SRMR
Model 1	161.45(183)	0.99	0.98	0.08		0.03
Model 2	841.20(186)	0.78	0.75	0.14	679.75 *** (3)	0.15
Model 3	1316.35(188)	0.62	0.57	0.18	1154.90 *** (5)	0.19
Model 4	1847.85(189)	0.44	0.37	0.21	1686.40 *** (6)	0.22

Note. N = 194. Model 1 = the four-factor model that included servant leadership (SL), perceived leader warmth (PLW), perceived leader competence (PLC), and leader effectiveness (LE). Model 2 = the three-factor model (PLW and PLC were combined). Model 3 = the two-factor model (SL, PLC, and PLW were combined). Model 4 = the one-factor model (SL, PLC, PLW, and LE were combined). *** *p* < 0.001 (two-tailed).

**Table 2:** Descriptive statistics and correlations in Study 2.

Variables		Mean	SD	1	2	3	4	5	6	7	8	9
1	Employee age	31.9	7.73									
2	Employee gender	0.50	0.50	−0.05								
3	Employee educational level	2.93	1.46	−0.03	0.00							
4	Employee tenure	1.94	1.67	0.07	−0.05	0.02						
5	Servant leadership	4.53	1.36	0.08	0.05	0.02	−0.03	**(0.95)**				
6	Leader gender	0.59	0.49	−0.04	−0.05	0.12	−0.02	0.05				
7	Perceived leader warmth	3.84	0.71	0.05	−0.12	0.06	0.04	0.38 **	−0.08	**(0.90)**		
8	Perceived leader competence	3.33	0.44	0.09	−0.07	−0.02	−0.09	0.03	0.02	0.08	**(0.93)**	
9	Leader effectiveness	3.99	0.46	0.13	−0.05	−0.20 **	−0.06	0.02	−0.08	0.22 **	0.21 **	**(0.91)**

Note. N = 194. Cronbach’s alphas presented on diagonal in bold where applicable. Leader gender and employee gender were coded 1 = male and 0 = female. ** *p* < 0.01 (two-tailed).

**Table 3:** Path analysis in Study 2.

Predictors	Perceived Leader Warmth		Perceived Leader Competence		Leader Effectiveness	
	B (SE)	*p*	B (SE)	*p*	B (SE)	*p*
**Control variables**						
Employee age	−0.00(0.01)	0.937	0.00(0.00)	0.431	0.01(0.00)	0.077
Employee gender	−0.19(0.10)	0.053	−0.02(0.06)	0.798	−0.01(0.07)	0.853
Employee educational level	0.02(0.03)	0.541	0.00(0.02)	0.858	−0.06(0.02)	0.009
Employee tenure	0.02(0.03)	0.475	−0.02(0.02)	0.193	−0.02(0.02)	0.326
**Independent variables**						
Servant leadership	0.15(0.06)	0.007	−0.11(0.04)	0.002	−0.04(0.03)	0.152
Leader gender	−0.49(0.35)	0.164	−0.89(0.22)	<0.001		
Interaction term						
Servant leadership × leader gender	0.08(0.07)	0.29	0.20(0.05)	<0.001		
**Mediators**						
Perceived leader warmth					0.16(0.05)	0.001
Perceived leader competence					0.15(0.08)	0.044

Note. N = 194. For gender, 1 = male, 0 = female. Coefficient = Unstandardized path coefficients. SE: standard error.

## Data Availability

The data presented in this study are available upon request from the corresponding author.

## Appendix A. Manipulation Materials in Study 1

**High Servant Leadership Condition**. In this simulation, imagine you have been working on a consulting team for 6 months. The team consists of the team leader Li Gang/Li Ting and three team members—you, Chen Ming and Zhao Yue. Since you started in your position, you have experienced the following in your work environment. The father of your coworker, Zhao Yue, broke his arm and needed some extra help during a few weeks of recovery. Your supervisor Li Gang/Li Ting allowed Zhao Yue to work some flexible hours during that time. Four months ago, a new project became available which you knew would be a good career-related experience for you, but you were on another project which better fit your current skills. You asked Li Gang/Li Ting to be moved to the new project even though it meant that you would be working more slowly until you learned the new set of skills. You were happy, but not surprised when you were allowed to move to the new project as that is commonly the way such requests are handled on your team. Not long after you started working on the new project, your team encountered an unexpected challenge that threatened to delay the completion date. Li Gang/Li Ting was quick to recognize that there was a problem with the project even though your team had not yet fully understood the fact that the project had encountered a major problem. Despite the high-profile nature of the project, Li Gang/Li Ting showed confidence in the team by empowering the team to find and implement your team’s solution for the problem. Again, you were not surprised as this is what your team has grown to expect from Li Gang/Li Ting. Twice during the past few months, Li Gang/Li Ting has had the opportunity to meet short term goals by making ethically questionable decisions. In both cases Li Gang/Li Ting clearly refused to bend any ethical rules, setting a good example for your team. In addition to your regular job, Li Gang/Li Ting encourages each team member to spend time volunteering for causes that give back to the community, even if those volunteer opportunities are small or unrelated to official corporate programs.

**Low Servant Leadership Condition**. In this simulation, imagine you have been working on a consulting team for 6 months. The team consists of the team leader Li Gang/Li Ting and three team members—you, Chen Ming and Zhao Yue. Since you started in your position, you have experienced the following in your work environment. You have noticed that your supervisor Li Gang/Li Ting is usually fair, but that decisions are made to maximize how upper management views the productivity of the team and, by extension, Li Gang/Li Ting. Sometimes this makes team members look less productive to upper management, but Li Gang/Li Ting does not believe that personal or professional concern for team members should get in the way of meeting group performance benchmarks. Four months ago, a new project became available that you knew would be a good career-related experience for you, but you were already on another project which better fit your current skills. You asked Li Gang/Li Ting to be moved to the new project, but Li Gang/Li Ting kept you on the old project because it would hurt the company if you worked more slowly as you tried to learn the skills needed for the new project. More recently, your team started to encounter a number of work-related problems that threatened to seriously delay a delivery deadline. Initially, Li Gang/Li Ting failed to recognize that something was going wrong at work. Then, after you explained the problems and suggested some solutions, Li Gang/Li Ting refused to let you handle the situation in your own way, telling you what to do instead. As part of the solution, Li Gang/Li Ting lied to the client about what your team was delivering to them. Although this tactic did result in successfully meeting the deadline, you believe that it did not adhere to the ethics training that you regularly receive. In addition to your regular job, you would like to spend time volunteering for causes that give back to the community. However, Li Gang/Li Ting was not supportive and told you that those volunteer opportunities were small or unrelated to official corporate programs.
